# Temporal Segregation between Dung-Inhabiting Beetle and Fly Species

**DOI:** 10.1371/journal.pone.0170426

**Published:** 2017-01-20

**Authors:** Frantisek Xaver Jiri Sladecek, Simon Tristram Segar, Colin Lee, Richard Wall, Martin Konvicka

**Affiliations:** 1 Faculty of Science, University of South Bohemia, Ceske Budejovice, Czech Republic; 2 Institute of Entomology, Biology Centre of the Academy of Science of the Czech Republic, Ceske Budejovice, Czech Republic; 3 School of Biological Sciences, University of Bristol, Bristol, United Kingdom; University of Pretoria, SOUTH AFRICA

## Abstract

The coexistence of ecologically similar species (i.e. species utilizing the same resource) is a major topic in ecology. Communities are assembled either through the biotic interactions of ecologically similar species, e.g. competition, or by the abiotic separation of species along gradients of environmental conditions. Here, we investigated the temporal segregation, succession and seasonality of dung-inhabiting Coleoptera and Diptera that utilize an identical resource in exactly the same way. The data were collected from two temperate pastures, one in the United Kingdom and the second in the Czech Republic. There was no evident temporal separation between ecologically similar coleopterous or dipterous taxa during succession. In contrast, these two orders were almost perfectly separated seasonally in both combined and site-specific datasets. Flies were most abundant in the summer, and beetles were more abundant in the spring and autumn. Ecologically similar beetles and flies also displayed seasonal separation in both combined and site-specific data. Analyses within site-specific data sets revealed such a separation at both the order and species level. Season is therefore the main temporal axis separating ecologically similar species of dung-inhabiting insects in temperate habitats, while succession aggregates species that may have similar environmental tolerances (to e.g. dung moisture). This separation between ecologically similar taxa of beetles and flies may be attributable to either competition-based niche separation or to temperature tolerance-based habitat filtering, since flies have peak activity in warmer months while beetles have peak activity in cooler months.

## Introduction

One of the most important questions in ecology is why there are communities and not single species assemblages [1]; the coexistence of species rich communities has been a topic of major research interest in ecology.

Natural communities are assembled, and species coexistence is facilitated, by two contrasting processes: niche differentiation and habitat filtering [2–4]. Niche differentiation separates species with similar traits and promotes the coexistence of ecologically similar species via their segregation [3, 5, 6]. Such segregation usually takes place along resource, habitat and temporal axes [7]. Habitat filtering aggregates species with similar traits, which in turn must have some tolerance to the environmental factors, such as temperature and humidity, etc. [2, 4, 6]. In addition to such ecological patterns, community assembly can also be detected in the phylogenetic structure of a community: if related species with similar traits are clustered it suggests habitat filtering; and if related species with similar traits are dispersed, it suggests niche differentiation [8, 9]. Although both habitat filtering and niche differentiation are quite extensively studied, there is still no consensus on the relationship between the mechanisms, nor which one is generally the most important for the coexistence of species rich communities [10–12]. However, environmental filtering is usually considered to be the main mechanism on large spatial scales, i.e. the assembly of large species rich communities [13–17], while both environmental filtering and niche differentiation are considered to play a role at the smaller spatial scale, e.g. relations between several species within the community [18, 19]. Niche differentiation and habitat filtering have usually been tested in communities present on multiple sites [e.g. 20] as well as in communities along altitudinal [e.g. 15, 21] or temporal gradients [e.g. 12].

Temporal gradients have generally been considered to play a key role in species coexistence (by separating similar competitors), although habitat filtering is probably also involved [5, 7, 12]. Temporal segregations usually involve the daily activity of ecologically similar species in actively foraging animals [18, 19]. Plants and “plant-like” sessile animals are separated along long-lasting successional gradients with competitively inferior species in early succession, and competitively dominant species in late succession, or vice versa [12, 22]. Finally, predominantly insect or invertebrate species coexistence is greatly maintained via their seasonal displacements [23, 24]. A good model system with species temporal patterns along all three gradients (daily activity, succession and seasonality), and in which both habitat filtering and niche separation could apply, are the communities inhabiting ephemeral habitats.

Ephemeral habitats, such as dung, carrion, fruiting bodies of mushrooms or rotten fruits, are temporally unstable, spatially and temporally random, yet usually provide a very high energy content for their inhabitants [25]. These inhabitants include fungi, bacteria and several animal groups, predominantly arthropods, nematodes and annelids [26, 27]. Temporal patterns of colonization of such habitats have been studied almost exclusively in insect representatives of Arthropods (all occupancy patterns). The temporal segregation among insects, at all three levels, is traditionally considered to be associated with niche differentiation due to instability and the limited mass of their primary resource [28, 29]. However, there is evidence for potential habitat filtering: for succession, for example mediated through dung moisture tolerance [30], and on the seasonal scale, for example mediated through temperature [31, 32] or drought tolerance [33]. The majority of such evidence comes from the insect community inhabiting the dung, which is the focus of the current study.

Competition and coexistence, based on niche differentiation in time, is very often cited for maintaining communities of dung-inhabiting (coprophilous) insects along temporal gradients [34]. However, the vast majority of studies that predict niche differentiation, especially in temperate communities, have been carried out solely using coprophilous beetles as the model communities, disregarding coprophilous flies [e.g. 24, 35]. Without flies–the second most prominent dung-inhabiting insect group–it is not possible to correctly assess whether the beetle temporal patterns are really due to their suggested niche differentiation with flies [36], or due to habitat filtering based upon dung pat properties for succession [e.g. 37].

The temporal patterns of beetles have been studied extensively and quantitatively both in temperate [e.g. 24, 37] and sub-tropical and tropical regions [e.g. 28, 38]. However, in contrast to beetles, fly temporal patterns have been studied quantitatively only infrequently [39, 40] and in a rather qualitative manner only [41, 42].

Here we therefore present a study focused on both dung-inhabiting beetles and flies, including both adults and larvae. We studied the two most prominent temporal segregations in temperate communities, succession and seasonality, using data from two sites, Central Europe [24, 40] and the United Kingdom [39]. We investigated: A) temporal patterns of the coprophilous beetles and flies as whole taxonomic groups, and B) temporal patterns of similar functional groups of coprophilous beetles and flies. Based on these patterns we further assessed whether the taxonomic or functional groups display niche differentiation (avoiding each other along the temporal gradient, with their model-fitted curves non-overlapping) or habitat filtering (co-occurrence along the temporal gradient, with their model-fitted curves overlapping).

## Material and Methods

### Study sites

The study was carried out on two pastures; one situated 10 km west of Ceske Budejovice, Czech Republic (CZ); and one situated 20 km south-west of Bristol, United Kingdom (UK).

Both pastures hosted a permanent herd of adult cows and had been continuously grazed in previous years. The CZ site is situated at 380 m.a.s.l., in a region with a mean annual temperature of 8.1°C, mean annual precipitation of 620 mm. The UK site is situated 100 m.a.s.l., in a region with a mean annual temperature of 11°C, mean annual precipitation of 850 mm. The UK site represents an oceanic climate with cooler summers and cool, yet not cold winters; the CZ site represents a continental climate with warm summers and colder winters. At both sites, the highest temperatures occur during the summer months (June–August) and the vegetation season spans from early spring (March–April) to autumn (October).

No official permit was required to carry the study on neither of study sites, as both are personal property of their owners. No special permit was needed to work with studied animals, as we worked with insects. One CZ beetle species, *Emus hirtus* (Linnaeus, 1758), is considered endangered in Czech Republic, however, we specifically did not killed individuals of this species (and immediately released them), as this species is easy to identify even in field.

### Insect sampling

At both sites, insect sampling was conducted using artificially created dung pats. Pats of 1.5 litres in volume were used in CZ, and pats of 1.5 kg of wet weight in the UK. The fresh, just defecated, dung was gathered from permanently stalled cows in CZ, and from pasture grazed cows after milking in the UK. Dung was thoroughly mixed and homogenized before exposition. The dung pats used for sampling were then created at the study sites. Following [43], we presume that there might be some minor differences between communities in dung from pasturing cows and communities in dung from cows fed on hay or silage, but no insect species was found to be exclusive to one of those types and super-abundant species were super-abundant in both such dung types [43]. We also presume that the artificially created dung pats should not substantially differ in their insect communities from pats naturally dropped by cows [43].

The CZ insect data were collected between 2009 and 2012 as part of three separate projects. The Coleoptera were sampled five times per vegetation season (11–29 April, 17 May– 4 June, 4–22 July, 15 August– 2 September and 23 September– 11 October) in 2009. During those seasons, the beetles were sampled from dung pats aged 1, 2, 3, 4, 5, 7, 9, 11 or 14 days. Each successional time was represented by one unique dung pat in each season.

Each successional time was replicated five times per season. So one replication consisted of a nine dung pats placed as a line in the field (nine successional times) with pats 5 m apart. The position of individual successional pats was randomized within this line. On the next day, another line (the second replication) was laid 5m apart from the first line (the first replication). On the third day three more replications were laid, forming a total of five replications per sampling season [24].

Adult Diptera were sampled in six sampling seasons: three in 2011 (23 April– 1 May, 16–24 July, 26 August– 3 September) and three in 2012 (9–17 May, 27 July– 4 August 2012, 14–22 September). The adult dipteran community was sampled at 1, 2, 3, 5, 7, 24, 27, 30, 48, 72, 96, 120, 144, 168 and 192 hours of dung pat age (first day, 1, 2, 3, 4, 5, 7 and 8 days of dung pat age). This sampling was conducted from five dung pats (each representing one replication per sampling season) [40].

Larval Diptera and Coleoptera were sampled three times in 2011 (18 April– 3 May, 12–27 July and 22 August– 6 September), from the pats aged 2, 3, 4, 5, 6, 7, 8, 9, 10, 11 and 12 days, each replicated four times per sampling season with identical pattern of replicate lines being laid on successive days as for CZ adult beetles. Data on fly larvae come from an unpublished study currently under review.

The CZ Coleoptera and larvae were sampled by floating the dung pat and the portion of underlying soil in a bucket of water. The CZ adult flies were sampled by rapidly covering the dung pat by a sweeping net, catching the disturbed fly individuals perching on the dung pat’s surface.

The UK data were collected in 2001 over a period of 24 weeks (the first week starting on 21 May, the last one on 29 October). At the start of each week, 10 dung pats were created and exposed in a pasture for 7 days, after which the pats were taken to the laboratory for insect extraction. Each dung pat was put in a fine-meshed bag and left 10 weeks to allow for the emergence of insects. As they emerged, live insects were funnelled into a collecting pot, to prevent the re-colonization of pats [39].

More details on the data sampling are provided in the respective publications [24, 39, 40].

### Functional groups and guilds

In addition to analyzing the raw numbers of beetles and flies, both beetle and fly species were classified into functional groups. For this, insect individuals were identified as further into species level as it was possible. Whenever species identification was not possible, we tried to establish the morpho species at lowest taxonomic level possible, which allowed for proper ecological classification.

The beetle and fly species were sorted primarily into three major functional groups: 1) the strict saprophages (i.e. coprophages), whose adults and larvae both utilise decaying matter or do not require living food to finish their development [44, 45]; 2) the omnivores, species which shift from predation to coprophagy (or vice versa) during their development [44, 46] or species that are not able to complete their development without living food even though their other development stages are coprophagous [45]; and 3) the predators, who utilise solely living prey when both larvae and adults [44, 47].

The saprophages could be further separated into two guilds that differ in their resource utilisation. Those are: i) the relocators, whose larvae do not live in the dung pat [24] and either live in underground nests prepared by their parents [48], or whose larvae utilise other kinds of decaying matter whilst their parents visit the dung pats solely for their own nutrition [49]. The second guild are: ii) the dwellers, whose larvae develop in the dung pat itself in at least one stage of their development. This guild comprises the majority of coprophilous saprophages [45, 49]. A detailed ecological classification of the beetle and fly taxa sampled is provided in the Supporting Information (S1 Table).

### Data preparation and taxa selection

The datasets (CZ and UK) were adjusted for two types of analyses; the site-specific analyses to compare the patterns between the two independent European temperate sites (CZ and UK), and one with the combined datasets to present the patterns at a broader temperate scale.

To make datasets from both sites comparable, the UK data were adjusted to the CZ data. The seasonal gradient in the CZ data is based primarily upon “seasons”, i.e. periods of year which are set up loosely at the month level, as starting and end dates are affected by weather conditions (early spring = April–first half of May; late spring = second half of May–June; early summer = July; late summer-early autumn = August–early days of September; and late autumn = second half of September–October). These seasons host a specific, yet predictable community composition (i.e. the dung insect community in early spring 2009 was almost identical to early spring 2011) as the seasonal patterns seem to be stable in both dung-inhabiting beetles [24, 39, 50, e.g. 51] and flies [40–42]. To match this seasonal pattern in the UK data, the weeks that overlapped with sampling seasons in CZ were chosen for future analyses (there were three UK weeks per one CZ sampling season). Since it was not possible to sample the early spring season in the UK, the early spring was also omitted from CZ data for the combined UK and CZ dataset, creating a dataset with 4 seasons. This CZ early spring was however used for analyses of individual sites, since otherwise the CZ site would have only two seasons for larval data. The arrangement of the UK dataset for single-site analyses was identical to that used for the combined data analysis.

The data from both sites, CZ and UK, were used to analyze the seasonal segregations between beetles and flies. However, only the data from the CZ site were used to investigate the successional separations, since the UK data were not sampled as successional lines.

Finally, the flies were represented by their larvae only in analyses along the successional gradient, whereas both fly adults and larvae were used in analyses along the seasonal gradient. This change was necessary because the beetle community, along the gradient of dung pat ageing, is much more likely to interact with fly larvae who inhabit the same interior of the dung pat, rather than with fly adults who perch on the dung pat surface and are most abundant only during the very first hours of dung pat existence [41, 42]. In the same fashion, only larvae of omnivorous beetles were used for analyses of successional segregation, since the vast majority of adult omnivorous beetles are very early successional and therefore do not interact with their larval fly omnivorous counterparts [24].

### Statistical analyses

The temporal trends of raw counts of beetles and flies, functional groups and ecological guilds of beetles and flies were analyzed using generalized linear models (GLM) in CANOCO 5 [52]. The response of each investigated group, e.g. beetle saprophages, was chosen as either linear or quadratic by AIC. To avoid the impact of over-dispersion, which is frequently present in GLMs with Poisson distribution of errors, all models were fitted with quasi-Poisson distribution of errors. The fitted GLM curves show us whether the beetle and fly functional groups 1) co-occur along temporal gradients (i.e. habitat filtering) [2], or 2) are separated (i.e. *a priori* niche differentiation) [3].

To further support the temporal trends of beetle and fly functional and ecological groups, we also analyzed the seasonal segregations among individual species of beetles and flies. We have chosen season exclusively, as we lack data on succession for the UK. Seasonal patterns of individual species were analyzed separately for the CZ and UK data with Detrended Canonical Correspondence Analysis (DCCA) in CANOCO 5 [52]. DCCA is a multivariate method suitable for data with unimodal species' responses along the gradient [53]. DCCA also prevents the occurrence of a prominent artifact, the arch effect (which occurred in our data when using non-detrending analysis like Canonical Correspondence Analysis). We used detrending by second order polynomial. Species data were log (x+1) transformed prior to the analyses. The significance of seasons, in the form of factorial variables, was tested by Monte Carlo permutation test (999 permutations).

## Results

The entire CZ dataset comprised 58,774 individuals in 107 species and morpho-species, the entire original UK dataset comprised 145,454 individuals in 47 species and morpho-species. Such a huge abundance of insects in the UK data was largely due to one species, *Sylvicola punctata* (Diptera: Anisopodidae), which was *a priori* omitted as an outlier, contributing 92,485 individuals in the UK dataset (64%). The combined dataset from both sites, after adjustments and omitting *S*. *punctata*, contains 78,036 individuals (28,645 UK; 49,391 CZ–excluding the early spring season) (Table 1). The datasets for individual sites consist of 28,645 individuals for the UK and 58,774 for CZ (including the early spring season).

**Table 1:** Summary of individual functional group abundances in the combined seasonal data and in the successional data (from Czech Republic).

**Beetles**	Late spring	early summer	late summer	autumn	sum of seasons	succession (CZ only)
all	12163	6341	10240	9263	38007	39810
saprophages (all)	3117	1140	935	2983	8175	11653
saprophages (dwellers)	1811	1107	830	2846	6594	7537
saprophages (relocators)	1306	33	105	137	1581	4116
omnivores/larvae	6404	4226	7554	4263	22447	218
predators	2642	975	1751	2017	7385	-
**Flies**	Late spring	early summer	late summer	autumn	sum of seasons	succession (CZ only)
all/larvae	3175	14657	13599	8598	40029	5385
saprophages (all)/larvae	2928	14175	13053	8039	38195	5263
saprophages (dwellers)/larvae	2836	13847	12941	7985	37609	5263
saprophages (relocators)	92	328	112	54	586	-
omnivores/larvae	245	379	544	559	1727	122
predators	2	103	2	0	107	-

Seasons: late spring = second half of May—June; early summer = July; late summer—early autumn = August—early days of September; and late autumn = second half of September—October. Functional groups: all = all beetles or flies, saprophages = both adults and larvae coprophagous (dwellers = larvae develop in the dung pats, relocators = larvae develop outside of the dung pat), omnivores = trophic switch between adults and larvae (usually adult saprophage, larva predator), predator = both adult and larvae predatory).

Since almost all GLMs were significant, we provide the significances and test-values of GLMs testing the dung-inhabiting insects trends in succession and seasonality in Table 2.

**Table 2:** Results of GLMs testing the seasonal and successional trends of coprophilous insects.

data	temporal gradient	order	functional group (guild)	df	*F*-value	*P*-value
combined	season	beetles	all	3 / 760	12.0	<10^−5^
combined	season	beetles	saprophages (all)	3 / 760	42.9	<10^−5^
combined	season	beetles	saprophages (dwellers)	3 / 760	42.5	<10^−5^
combined	season	beetles	saprophages (relocators)	3 / 760	40.6	<10^−5^
combined	season	beetles	omnivores	3 / 760	2.1	0.13
combined	season	beetles	predators	3 / 760	27.4	<10^−5^
combined	season	flies	all	3 / 760	17.1	<10^−5^
combined	season	flies	saprophages (all)	3 / 760	17.8	<10^−5^
combined	season	flies	saprophages (dwellers)	3 / 760	17.9	<10^−5^
combined	season	flies	saprophages (relocators)	3 / 760	7.2	<10^−3^
combined	season	flies	omnivores	2 / 761	16.6	<10^−4^
combined	season	flies	predators	3 / 760	29.4	<10^−5^
CZ	season	beetles	all	3 / 744	4.3	0.01
CZ	season	beetles	saprophages (all)	3 / 744	24.6	<10^−5^
CZ	season	beetles	saprophages (dwellers)	3 / 744	24.4	<10^−5^
CZ	season	beetles	saprophages (relocators)	2 / 745	79.7	<10^−5^
CZ	season	beetles	omnivores	2 / 745	4.5	0.04
CZ	season	beetles	predators	3 / 744	10.3	<10^−4^
CZ	season	flies	all	3 / 744	25.3	<10^−5^
CZ	season	flies	saprophages (all)	3 / 744	25.9	<10^−5^
CZ	season	flies	saprophages (dwellers)	3 / 744	25.3	<10^−5^
CZ	season	flies	saprophages (relocators)	3 / 744	6.5	<10^−2^
CZ	season	flies	omnivores	2 / 745	6.2	0.01
CZ	season	flies	predators	3 / 744	16.0	<10^−5^
UK	season	beetles	all	3 / 117	23.0	<10^−5^
UK	season	beetles	saprophages (all)	2 /118	4.0	0.05
UK	season	beetles	saprophages (dwellers)	2 /118	3.9	0.05
UK	season	beetles	saprophages (relocators)	3 / 117	15.2	<10^−5^
UK	season	beetles	omnivores	3 / 117	28.5	<10^−5^
UK	season	beetles	predators	3 / 117	13.0	<10^−5^
UK	season	flies	all	3 / 117	80.4	<10^−5^
UK	season	flies	saprophages (all)	3 / 117	73.6	<10^−5^
UK	season	flies	saprophages (dwellers)	3 / 117	73.6	<10^−5^
UK	season	flies	omnivores	3 / 117	23.4	<10^−5^
UK	season	flies	predators	3 / 117	48.3	<10^−5^
CZ	succession	beetles	all	3 / 744	5.0	<10^−2^
CZ	succession	beetles	saprophages (all)	3 / 744	5.7	<10^−2^
CZ	succession	beetles	saprophages (dwellers)	3 / 744	10.7	<10^−4^
CZ	succession	beetles	saprophages (relocators)	2 / 745	10.4	<10^−2^
CZ	succession	beetles larvae)	omnivores	3 / 744	38.3	<10^−5^
CZ	succession	flies (larvae)	all	3 / 744	9.3	<10^−4^
CZ	succession	flies (larvae)	saprophages (all)	3 / 744	9.1	<10^−3^
CZ	succession	flies (larvae)	omnivores	3 / 744	26.1	<10^−5^

data = from which data set the trend has been calculated (CZ = Czech Republic data, UK = United Kingdom data, combined = CZ+UK), temporal gradient = which temporal gradient was used as the environmental variable (season or succession), order = the model was fitted for either beetles or flies, functional group = for which functional group the model was fitted (all = all beetles or flies, saprophages = both adults and larvae coprophagous (dwellers = larvae develop in the dung pats, relocators = larvae develop outside of the dung pat), omnivores = trophic switch between adults and larvae (usually adult saprophage, larva predator), predator = both adult and larvae predatory), df = degrees of freedom (1 dung pat = 1 observation) of that particular GLM in format: model used df / residual df (model df 2 = linear curve, model df 3 = quadratic curve).

### Temporal patterns of coprophilous beetles and flies as general taxonomic groups

**Fig 1:**
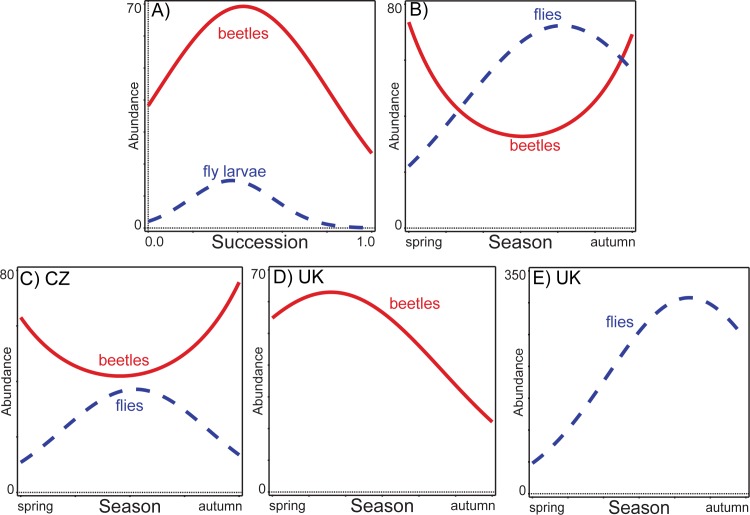
Trends of dung-inhabiting beetle and fly abundances during succession (Czech Republic) and season (both sites). A) successional trends of dung-inhabiting beetles (solid red line) and fly larvae (dashed blue line) (data from the Czech Republic), B) seasonal trends of dung-inhabiting beetles (solid red line) and flies (dashed blue line) (data from both sites), C) seasonal trends of dung-inhabiting beetles (solid red line) and flies (data from the Czech Republic), D) seasonal trends of dung-inhabiting beetles (data from the United Kingdom), E) seasonal trends of dung-inhabiting flies (data from the United Kingdom),

Beetles and flies displayed significant trends along both the successional and seasonal gradients (Fig 1A and 1B). Both of these temporal gradients, however, strongly differed. Along the successional gradient, the occurrence of beetles and fly larvae almost overlapped, with both groups peaking at practically the same time; while with respect to season beetles and flies displayed a separation in the combined data. Flies reached their maximum abundance during summer, especially late summer, while beetles were most abundant during the spring and autumn seasons.

The pattern of seasonal displacement between beetles and flies was also seen at both individual sites (Fig 1C, 1D and 1E), with beetles being abundant in spring–autumn while flies were most abundant in summer in CZ, and with beetles being the most abundant in the earlier part of the season (spring), while flies were the most abundant in the later part of the season (mostly late summer) in the UK.

### Seasonal-temporal patterns of beetle and fly functional groups

The majority of ecological groups displayed a significant trend along the seasonal gradient in the combined CZ+UK data and also in site-specific datasets (Table 2).

**Fig 2:**
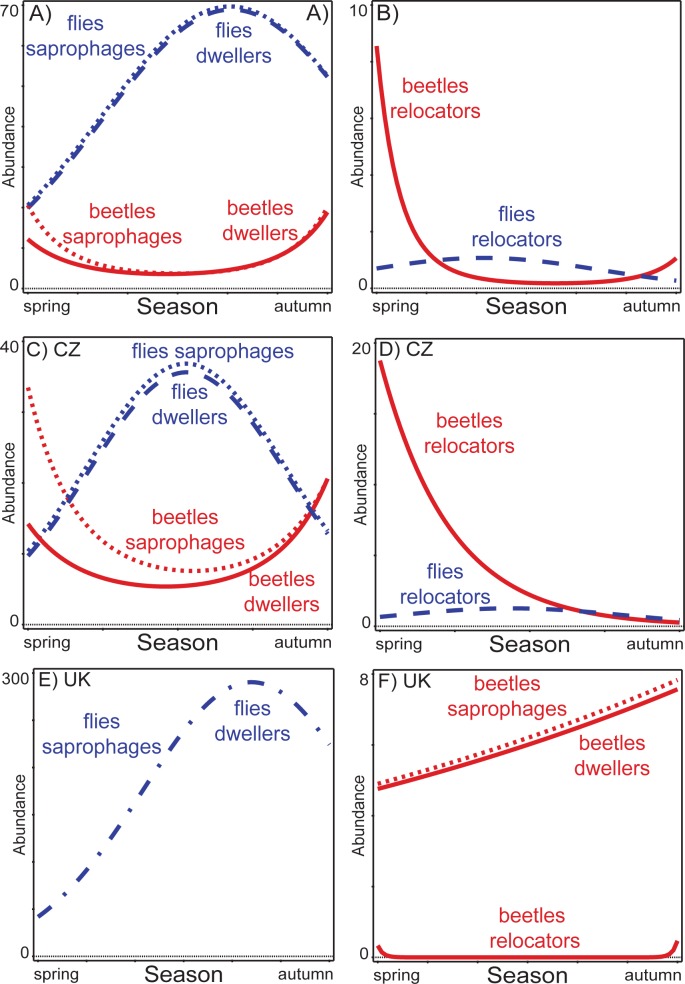
Seasonal trends of saprophagous dung-inhabiting beetles, flies and their guilds in the combined data and data from both sites. Seasonal trends are presented for the combined data (A, B), data from the Czech Republic (C, D) and data from the United Kingdom (E, F). The fly patterns are represented by blue lines, the beetle patterns are represented by red lines. Dwellers = species whose larva develop in the dung pat, relocators = species whose larva develop outside of the dung pat.

The saprophagous beetles in the combined data were clearly numerically separated seasonally from the saprophagous flies (Fig 2A), being most abundant during spring and autumn; while saprophagous flies were most abundant during the summer. The same pattern was also retrieved at both sites, especially at the CZ site (Fig 2C). The saprophagous beetles and flies were also separated in season at the UK site, but saprophagous beetles, although on the verge of significance (*P* = 0.05), increased their numbers in the later part of season when fly numbers were decreasing (Fig 2E).

The seasonal patterns of all saprophagous beetles and flies for both the combined and site-specific datasets were mimicked by the responses of the saprophagous beetle and fly dwellers guilds, as the majority of saprophagous beetles and flies were dwellers (Fig 2A, 2C and 2E).

The second saprophagous guild of relocators also displayed seasonal separation between beetle relocators, dominating spring and partly autumn, and fly relocators, dominating summer (Fig 2B) in combined data. A similar separation among beetle and fly relocators was also retrieved in the site-specific data (Fig 2D and 2F).

**Fig 3:**
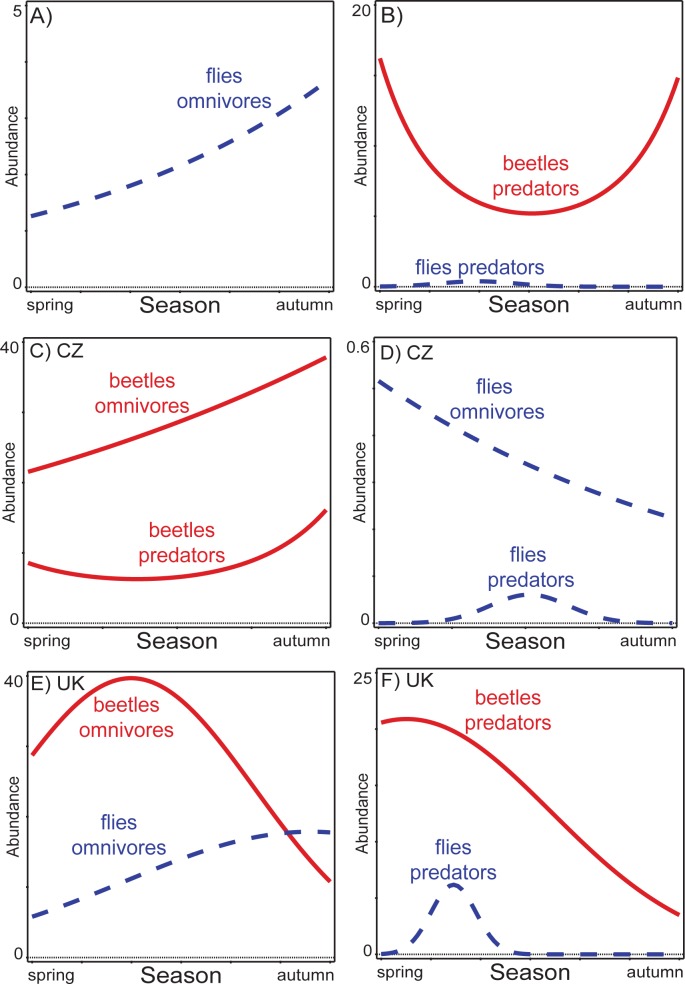
Seasonal trends of omnivorous and predatory dung-inhabiting beetles and flies in the combined data and the data from both sites. Seasonal trends are presented for the combined data (A, B), data from Czech Republic (C, D) and data from United Kingdom (E, F). The fly patterns are represented by blue lines, the beetle patterns are represented by red lines. Omnivores = trophic shift between adult and larva (adult usually saprophagous, larva predatory).

The omnivorous beetles did not display a significant seasonal trend in the combined data (*F* = 2.1, *P* = 0.13), while the number of omnivorous flies increased throughout the season (Fig 3A). In contrast, the omnivorous beetles and flies were seasonally separated at individual sites (Fig 3C, 3D and 3E).

Despite that the fact that predatory flies did not reach abundances comparable with predatory beetles (107 vs. 7385, see Table 1), they did display a clear separation from the predatory beetles by their short occurrence in early summer, while the numbers of predatory beetles were almost the lowest in the combined data (Fig 3B). A similar pattern also applied for both individual sites, where predatory flies displayed a peak of abundance while the predatory beetles had their minimum abundance in CZ (Fig 3C and 3D), or when the number of beetle predators were declining in the UK (Fig 3F).

### Seasonal-temporal patterns of beetle and fly species

**Fig 4:**
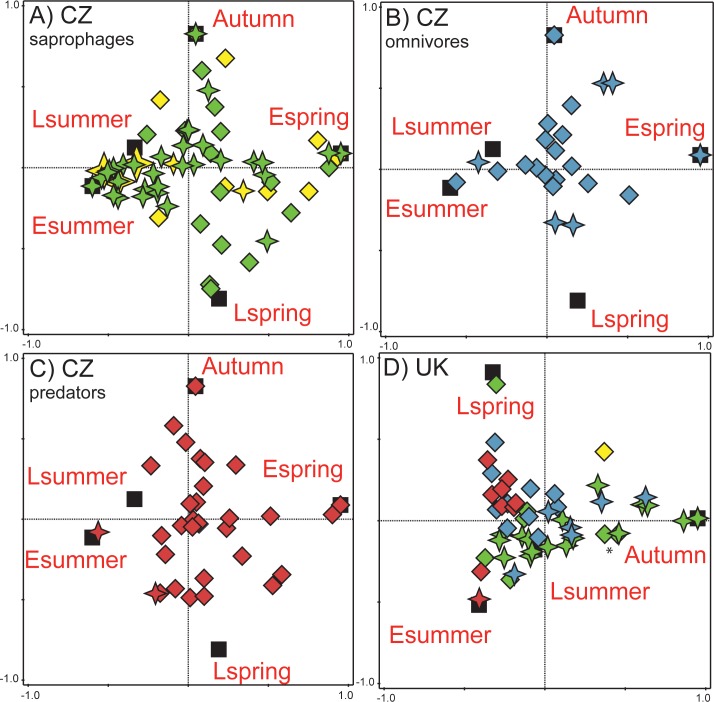
DCCA ordination diagrams of species seasonal segregation at both sites. Panels A, B and C represents the seasonal segregation of CZ species, all originating from one analyses (*F* = 12.6, *P* = 0.001). Those panels were created by including just one functional group for better visualization of results (A = saprophages (both adults and larvae coprophagous), B = omnivores (trophic switch between adults and larvae (usually adult saprophage, larva predator)), C = predators (= both adult and larvae predatory). Panel D represents the overall results of seasonal segregation in UK species (*F* = 17.3, *P* = 0.001). black squares = centroids of individual seasons (Espring = early spring (April–first half of May), Lspring = late spring (second half of May–June), Esummer = early summer (July), Lsummer = late summer (August–early days of September), Autumn = second half of September–October), diamonds = beetle species, stars = fly species, yellow symbols = relocators (larvae develop outside of the dung pat), green symbols = dwellers (larvae develop in the dung pats), blue symbols = omnivores, red symbols = predators, an asterisk (*) = the most abundant saprophage species in the UK data.

The species assemblages were significantly structured by season in both the CZ data (*F* = 12.6, *P* = 0.001, all axes explain 6.4% of variability in species data) and UK data (*F* = 17.3, *P* = 0.001, all axes explain 30.9% of variability in species data). In general, species seasonal patterns highly support the results of GLMs. This is most recognizable in the CZ beetle and fly saprophages, where beetle species almost exclusively preferred spring and late-summer/autumn seasons, while fly species greatly preferred early and late summer seasons (Fig 4A). In CZ omnivorous species, beetles reached their optima from the early summer to autumn with most species preferring the late summer/autumn season, while fly species were almost equally distributed between spring and autumn seasons (Fig 4B). Two thirds of CZ beetle predatory species reached their optima in either spring or late summer/autumn seasons with one third occurring between early and late summer (Fig 4C). On the other hand, CZ fly predators occurred almost exclusively in early summer or between late spring and early summer (Fig 4C). In the UK data, there were interchanges between late spring occurring beetle predators and early summer occurring fly predators, and again late spring occurring beetle omnivores and late summer/autumn occurring fly omnivores (Fig 4D). In saprophages, the majority of UK fly saprophages were associated with early/late summer, while some beetle species also occurred in the summer seasons, but the most abundant species had their optima in late summer/autumn seasons (Fig 4D).

### Successional-temporal patterns of beetle and fly functional groups

**Fig 5:**
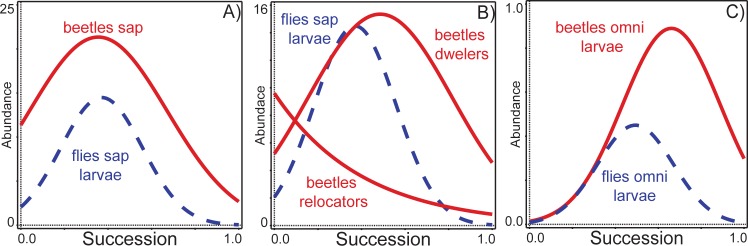
Successional trends in dung-inhabiting beetles and flies. A) beetle (solid red line) and fly larvae (dashed blue line) saprophages (sap); B) beetle relocators and dwellers (solid red lines) and fly larvae (dashed blue line) saprophages (sap); C) beetle omnivorous larvae (omni: solid red line) and fly omnivorous larvae (omni: dashed blue line).

The optima of both saprophagous beetles and saprophagous fly larvae almost overlapped along the successional gradient (Fig 5A). At the individual guild level, the larvae of saprophagous flies, represented exclusively by fly larvae dwellers, had their peak abundance slightly after the highest abundance of beetle relocators and almost together with the maximum abundance of beetle dwellers (Fig 5B). The larvae of omnivorous beetles had their maximum just slightly after the peak in abundance of omnivorous fly larvae (Fig 5C) (Table 1).

## Discussion

Herein we have shown that the coprophilous beetles and flies, as the main components of dung-inhabiting communities, display relatively robust temporal separation at both order and species level. In both the combined data from our sites and at individual sites, the periods of peak abundance of the coprophagous and predatory flies and beetles with similar ecology appear to avoid each other during the season (i.e. niche differentiation). The omnivorous beetles and flies were not temporally separated in the combined data, but they displayed a solid seasonal separation at individual sites at both order and species level. In contrast, the potential competitors from among the coprophagous and omnivorous functional groups co-occurred along the successional gradient (i.e. habitat filtering).

### Biotic and abiotic interpretations of temporal segregations

The seasonal separation between all beetles and flies, and especially among representatives of ecological groups could have two potential explanations. The temporal dynamics of the coprophilous insect temperate community reflect either the biotic interactions of its species, i.e. competition or predation (niche differentiation) [5, 7]; or they reflect the patterns of species adaptation to certain environmental conditions (habitat filtering) [2, 4].

The seasonal separation among both beetles and flies and their respective functional groups should indicate niche differentiation [3, 6]. The present temperate community of coprophilous insects should therefore be formed as a result of recent or historical competition, or even possibly intra-guild predation [54].

The asymmetrical competition between saprophagous beetles and flies is well-known, as large dung relocating beetles can quickly deplete dung for the dung dwelling fly larvae [55]. This competition is, however, restricted to regions with a dominant presence of such dung relocating beetles, i.e. the tropical, sub-tropical and Mediterranean regions [e.g. 56]. In contrast, the temperate beetle communities are comprised almost exclusively of beetles whose larvae also develop in the dung pats (dwellers) [49]. The relationship of those dwelling beetles and flies are, however, rather complex. The dwelling beetles could negatively affect fly larvae survival, by destroying their eggs [57]. On the other hand, beetles tend to avoid oviposition in the presence of high abundances of fly larvae [58]. Finally, if the fly larvae are excluded from dung pats, the saprophagous beetle adults and larvae abundances are affected negatively [59]. We therefore suggest that, given the current evidence, the temporal separation of temperate saprophagous beetles and flies cannot be satisfactorily explained by recent competition.

Historical competition between beetles and flies could be a viable explanation. Many species of the Czech and British coprophilous fauna probably dispersed from their Mediterranean glacial refugia [60–62] since they have ranges spanning from the Mediterranean region northward [63, 64]. In that case, the coprophilous insects could still follow the temporal patterns established in the Mediterranean region. Here the saprophagous beetles, especially the competitively dominant large dung relocating species also occur primarily in spring and partly in autumn [38, 56, 65]. Flies on the other hand should be most abundant during summer and early autumn [55, 66, 67].

Biotic interactions could also play an important role in facilitating the temporal patterns of omnivores and predators. In those two trophic groups, potential intra-guild predation could play the major role, since it can affect the predated species behavior or habitat choice [68–70]. In both such groups, the beetles presumably play the dominant role over the flies in the seasonal separation. In omnivores, the fly larvae are very similar to the larvae of other flies, having only different modification of their mouth hooks [71]. In contrast, the larvae of beetles have hard-biting mouthparts and are generally ferocious predators of fly larvae in general [46, 72]. Despite the fact that the larvae of predatory flies can kill the larvae of bark beetles [73], they probably cannot compete against the mobile and voracious larvae of predatory beetles, nor their adults.

Despite the fact that seasonal separation of coprophilous beetles and flies indicates niche differentiation, there is more speculation than actual evidence for real competitive segregation.

Habitat filtering on the other hand might also be a valid explanation for the beetle and fly seasonal patterns. Contrary to niche differentiation, habitat filtering aggregates species with similar traits, mostly because of their tolerances to the abiotic environmental factors–which should change along the seasonal gradient in our study [2, 4]. The most obvious, seasonally dependent, environmental factor in temperate environments is temperature.

The relations between beetles, flies and the ambient temperature have not been studied extensively; however, they could provide a simple interpretation for the seasonal displacement between those groups in temperate environments. Temperate dwelling saprophagous beetles should be susceptible to higher temperatures (>30°C), especially in larval stages [74, 75]. On the other hand, such beetles can be active in relatively low temperatures (5–10°C) [74]. The same principles probably apply also to omnivorous and predatory beetles, as well as to fly omnivores [41, 76]. In contrast, the majority of saprophagous flies need higher temperatures to become active (>10°C), but they are more resistant to higher temperatures in general [41]. In fact, higher temperatures enable their larvae to finish their development more quickly, avoiding potential predation [41]. In addition, the temperature-based separation of beetles and flies also applies in the Mediterranean region, since high temperatures could be lethal to dung relocating beetles if they do not possess any heat-regulating ability [31]. Finally, the seasonality based upon temperature tolerance would be a very simple explanation as to why the European species display an identical seasonal pattern in artificially-formed communities in North America in which the majority of species are immigrants from Europe [42, 77].

In contrast to the seasonal patterns, there were no temporal separations among the species utilizing the resource in the same way during the succession of coprophilous beetles and flies. Successional patterns in this community indicate *a priori* habitat filtering, in which species with similar ecology tend to aggregate in the succession, probably along with favorable chemical or moisture conditions [30, 37].

### Temporal trends in dung and other systems

We find environmentally-based filtering to be the most parsimonious explanation for the assembly of the temperate coprophilous insect community, as it is probably driving both the successional and seasonal gradients. Our findings agree with the prediction [13–17] that environmental filtering is the main community assembly mechanism, applying also in dung and in other insect communities inhabiting other ephemeral habitats [78, 79].

On the other hand, the niche-based separations among coprophilous insects and insects inhabiting similar ephemeral habitats [23, 80] could take place on the smaller scale of individual sites. For example, even if coprophagous flies and beetles are seasonally separated, the coprophagous representatives of both groups are temporally aggregated and co-exist probably in niche-based separation [34], i.e. macro- (e.g. forest vs. open field) or microhabitat (dung type, dung pat size) preferences.

### Site differences, study limitations and future suggestions

Both the combined and site-specific data depicted the main result as seasonal separation between coprophilous beetles and flies, and their respective functional groups. The site-specific data differed sometimes slightly in the shapes of the seasonal patterns, e.g. omnivorous beetles were mostly sampled throughout the spring and partly in the summer in UK, while they were the most abundant in the autumn in CZ. Such differences could stem from the different sampling methods used at both sites, since the CZ data contain the community actually present in the dung pat, whereas the UK data present who is leaving the pat, including reared individuals. Taking omnivorous beetles as an example, they should be therefore most abundant in autumn (according to the CZ data), but reproduce more in spring (according to the UK data).

Our hypothesis that habitat filtering is the main assembly rule in coprophilous insects, and probably in other communities inhabiting ephemeral habitats, needs of course rigorous testing in further studies. We therefore suggest that the main pathways for future studies should include: 1) dung pat physical (e.g. moisture development) and chemical (e.g. dung volatiles, and other dung chemistry) attributes during succession (paired with testing the species’ affinity or resistance to them); 2) species’ temperature tolerances/affinities; or 3) detailed studies on the competitive and trophic interaction between beetle and fly species.

## Supporting Information

S1 Table Ecological classification of dung-inhabiting beetles and flies used in this study. Fun. group = functional group (saprophages = both adult and larvae saprophagous, omnivores = trophic shift between adult and larva (adult usually saprophagous, larva predatory), predators = both adult and larva predatory), dwellers = species whose larva develop in the dung pat, relocators = species whose larva develop outside of the dung pat, model representative = an example of a species belonging to that exact functional group/taxonomic group and was present in our sampling. If a taxonomic group was not identified beyond the genus level, the model representative is genus spp., if identification was not possible beyond the family level, no representative is given.
(DOC)
