# Supplementary material for: Temporal Segregation between Dung-Inhabiting Beetle and Fly Species
Source: PLoS One. 2017 Jan 20;12(1):e0170426. doi: 10.1371/journal.pone.0170426 (PMC5249136; doi:10.1371/journal.pone.0170426)
Supplement: S1 Table — Fun. group = functional group (saprophages = both adult and larvae saprophagous, omnivores = trophic shift between adult and larva (adult usually saprophagous, larva predatory), predators = both adult and larva predatory), dwellers = species whose larva develop in the dung pat, relocators = species whose larva develop outside of the dung pat, model representative = an example of a species belonging to that exact functional group/taxonomic group and was present in our sampling. If a taxonomic group was not identified beyond the genus level, the model representative is genus spp., if identification was not possible beyond the family level, no representative is given. (DOC) [file pone.0170426.s001.doc]

Table S1: Ecological classification of dung-inhabiting beetles and flies used in this study.

| **Order** | **Family** | **Sub-family** | **Fun. group** | **guild** | **model representative** |
| --- | --- | --- | --- | --- | --- |
| beetles | Carabidae | - | predators | - | *Amara aenea* (De Geer, 1774) |
| beetles | Geotrupidae | - | saprophages | relocators | *Geotrupes spiniger*  (Marsham, 1802) |
| beetles | Histeridae | - | predators | - | *Hister unicolor* Linnaeus, 1758 |
| beetles | Hydrophilidae | - | omnivores | - | *Sphaeridium bipustulatum* Fabricius, 1781 |
| beetles | Scarabaeidae | Aphodiinae | saprophages | relocators | *Aphodius erraticus* (Linnaeus, 1758) |
|  |  | Aphodiinae | saprophages | dwellers | *Aphodius ater* (De Geer, 1774) |
|  |  | Scarabaeinae | saprophages | relocators | *Onthophagus coenobita*  (Herbst, 1783) |
| beetles | Staphylinidae | Oxytelinae | saprophages | dwellers | *Anotylus rugosus* (Fabricius, 1775) |
|  |  | Proteininae | saprophages | dwellers | *Megarthrus depressus*  (Paykull, 1789) |
|  |  | other subfamilies | predators | - | *Philonthus cruentatus*  (Gmelin, 1790) |
| flies | Anthomyiidae | - | saprophages | - | - |
| flies | Calliphoridae | - | saprophages | relocators | *Lucilia casear* (Linnaeus, 1758) |
| flies | Carnidae | - | saprophages | dwellers | *Meoneura* spp. |
| flies | Chironomidae | - | saprophages | dwellers | - |
| flies | Chloropidae | - | saprophages | relocators | - |
| flies | Dolichopodidae | - | predators | - | - |
| flies | Empididae | - | predators | - | *Empis* spp. |
| flies | Fanniidae | - | saprophages | dwellers | *Fannia* spp. |
| flies | Hybotidae | - | predators | - | *Drapetis* spp. |
| flies | Limoniidae | - | saprophages | dwellers | *Rhipidia maculata* Meigen, 1818 |
| flies | Milichiidae | - | saprophages | dwellers | *Madiza glabra* Fallén, 1820 |
| flies | Muscidae | Muscinae | omnivores | - | *Mesembrina meridiana*  (Linnaeus, 1758) |
|  |  | Muscinae | saprophages | dwellers | *Musca autumnalis* De Geer, 1776 |
|  |  | Mydaeinae | omnivores | - | *Hebecnema vespertina*  (Fallén, 1823) |
|  |  | Mydaeinae | saprophages | relocators | *Myospila meditabunda*  (Fabricius, 1781) |
| flies | Sarcophagidae | - | saprophages | dwellers | *Ravinia pernix* (Harris, 1780) |
| flies | Scathophagidae | - | omnivores | - | *Scatophaga stercoraria*  (Linnaeus, 1758) |
| flies | Scatopsidae | - | saprophages | dwellers | - |
| flies | Sciaridae | - | saprophages | dwellers | - |
| flies | Sepsidae | - | saprophages | dwellers | *Saltella sphondylii* (Schrank, 1803) |
| flies | Sphaeroceridae | - | saprophages | dwellers | *Lotophila atra* (Meigen, 1830) |
| flies | Stratiomyidae | - | saprophages | dwellers | *Sargus flavipes* Meigen, 1822 |
| flies | Syrphidae | - | saprophages | dwellers | - |

Fun. group = functional group (saprophages = both adult and larvae saprophagous, omnivores = trophic shift between adult and larva (adult usually saprophagous, larva predatory), predators = both adult and larva predatory), dwellers = species whose larva develop in the dung pat, relocators = species whose larva develop outside of the dung pat, model representative = an example of species that belongs into that exact functional group/taxonomic group and was presented in our sampling. If a taxonomic group was not identified behind genus level, the model representative is genus spp., if identification was not possible behind family level, no representative is given.
